# Association between pain intensity and depressive symptoms in community-dwelling adults: longitudinal findings from the Survey of Health, Ageing and Retirement in Europe (SHARE)

**DOI:** 10.1007/s41999-023-00835-5

**Published:** 2023-07-14

**Authors:** Giulia Ogliari, Jesper Ryg, Karen Andersen-Ranberg, Lasse Lybecker Scheel-Hincke, Jemima T. Collins, Alison Cowley, Claudio Di Lorito, Vicky Booth, Roelof A. J. Smit, Ralph K. Akyea, Nadeem Qureshi, David A. Walsh, Rowan H. Harwood, Tahir Masud

**Affiliations:** 1 Department of Health Care of Older People (HCOP), Queen’s Medical Centre, Nottingham University Hospitals NHS Trust, Derby Road, Nottingham, Nottinghamshire NG7 2UH UK; 2 Department of Geriatric Medicine, Odense University Hospital, Odense, Denmark; 3 Geriatric Research Unit, Department of Clinical Research, University of Southern Denmark, Odense, Denmark; 4 Unit for Epidemiology, Biostatistics and Biodemography, Department of Public Health, University of Southern Denmark, 5000 Odense, Denmark; 5 University of Nottingham, Nottingham, UK; 6 Research & Innovation, Nottingham University Hospitals NHS Trust, Nottingham, UK; 7 Division of Primary Care and Population Health, University College London, London, UK; 8 Novo Nordisk Foundation Center for Basic Metabolic Research, Faculty of Health and Medical Sciences, University of Copenhagen, Blegdamsvej 3B, Building 7 (Maersk Tower), 2200 Copenhagen, Denmark; 9 Primary Care Stratified Medicine, School of Medicine, University of Nottingham, Nottingham, UK; 10 Pain Centre Versus Arthritis, University of Nottingham, Nottingham, UK; 11 Sherwood Forest Hospitals NHS Foundation Trust, Sutton-in-Ashfield, UK; 12 NIHR Applied Research Collaboration-East Midlands, Nottingham, UK

**Keywords:** Pain, Depressive symptoms, Loneliness, Population-based prospective study, Sex-differences, Ageing

## Abstract

**Purpose:**

To investigate the longitudinal associations between pain and depressive symptoms in adults.

**Methods:**

Prospective cohort study on data from 28,515 community-dwelling adults ≥ 50 years, free from depression at baseline (Wave 5), with follow-up in Wave 6 of the Survey of Health, Ageing and Retirement in Europe (SHARE). Significant depressive symptoms were defined by a EURO-D score ≥ 4. The longitudinal association between baseline pain intensity and significant depressive symptoms at follow-up was analysed using logistic regression models; odds ratios (ORs) and confidence intervals (CI) were calculated, adjusting for socio-demographic and clinical factors, physical inactivity, loneliness, mobility and functional impairments.

**Results:**

Mean age was 65.4 years (standard deviation 9.0, range 50–99); 14,360 (50.4%) participants were women. Mean follow-up was 23.4 (standard deviation 3.4) months. At baseline, 2803 (9.8%) participants reported mild pain, 5253 (18.4%) moderate pain and 1431 (5.0%) severe pain. At follow-up, 3868 (13.6%) participants—1451 (10.3%) men and 2417 (16.8%) women—reported significant depressive symptoms. After adjustment, mild, moderate and severe baseline pain, versus no pain, were associated with an increased likelihood of significant depressive symptoms at follow-up: ORs (95% CI) were 1.20 (1.06–1.35), 1.32 (1.20–1.46) and 1.39 (1.19–1.63), respectively. These associations were more pronounced in men compared to women, and consistent in participants aged 50–64 years, those without mobility or functional impairment, and those without loneliness at baseline.

**Conclusion:**

Higher baseline pain intensity was longitudinally associated with a greater risk of significant depressive symptoms at 2-year follow-up, in community-dwelling adults without baseline depression.

**Supplementary Information:**

The online version contains supplementary material available at 10.1007/s41999-023-00835-5.

## Key summary points

**Aim:**

To investigate the longitudinal association between pain intensity at baseline and risk of developing significant depressive symptoms, at 2-year follow-up, in community-dwelling adults aged ≥ 50 years, without depression at baseline, in the Survey of Health, Ageing and Retirement in Europe (SHARE).

**Findings:**

Higher pain intensity at baseline was associated with an increased risk of developing significant depressive symptoms in community-dwelling adults, at 2-year follow-up, independent of socio-demographic and clinical factors, physical inactivity, loneliness, mobility and functional impairments. This association was more pronounced in men compared to women.

**Message:**

Self-reported mild, moderate and severe pain, respectively, versus no pain were risk factors for onset of significant depressive symptoms in community-dwelling adults without depression at baseline.

**Supplementary Information:**

The online version contains supplementary material available at 10.1007/s41999-023-00835-5.

## Introduction

Depression is a common condition affecting about 300 million adults worldwide [1]. In Europe, it affects about 1–12% adults, with significant variations across countries [2–4]. Depression is a major cause of years lived with disability in adults [1]. It has been associated with a higher risk of myocardial infarction, heart failure, stroke and peripheral arterial disease and a higher risk of cardiovascular and all-cause mortality [5–9]. Epigenetic studies have shown that depression may accelerate biological ageing, including brain ageing [10]. Late-life depression is a risk factor for vascular dementia and Alzheimer’s disease [11]. Therefore, it is a public health priority to identify potentially reversible and common risk factors for depression, accounting for the role of multiple factors and pathways.

Depression has a multifactorial aetiology. The individual vulnerability to depression is moderated by the interplay of multiple protective and risk factors for depression [12]. Women are twice as likely as men to develop depression during both younger and older adulthood [13]. A few longitudinal studies, while not others, identified higher education as a protective factor against depression among adults, compared to lower education [12, 14]. Higher physical activity may also be a protective factor against depression in adults [12, 15]. Loneliness, defined as the discrepancy between one’s desired and one’s actual relationships, may lead to depression but the evidence is inconclusive [12, 16].

The prevalence of depression increases with age, but it is unclear whether older age itself is a risk factor for depression or whether the older age-depression association is explained by the higher prevalence of other risk factors for depression including co-morbidities and impairments in older adults. Among co-morbidities, stroke and arthritis have been longitudinally associated with a higher risk of depression [12]. Mobility impairment has been consistently associated with a higher incidence of depression, while functional impairments less consistently [12, 17].

A bidirectional association between pain and depression has been hypothesized [18]. Pain may lead to depression, while depression may precede and exacerbate pain. Pain more frequently occurs in adults with co-morbidities such as arthritis, a known risk factor for depression [12]. Pain has also been causally linked to physical inactivity, mobility and functional impairment, which in turn may favour the onset of depression [19, 20]. Pain may contribute to social isolation and loneliness, in both middle-aged and older adults [21–23]. To disentangle the association between pain and depression it is crucial to take into account a wide range of potential co-variates and mediators [24, 25].

Pain is common among adults and its prevalence has been increasing in both the U.S. and Europe in recent years [26, 27]. In a U.S. nationally-representative study, about half of adults ≥ 65 years reported pain in the previous month [28]. As with depression, pain is reported more frequently by women than men [29]. Given that pain is highly prevalent and potentially reversible, it is essential to further elucidate the link between pain and depression.

Therefore, we explored the longitudinal association between pain intensity at baseline and significant depressive symptoms at 2-year follow-up in community-dwelling participants aged ≥ 50 years, without depression at baseline, adjusting for sociodemographic and clinical factors, in the Survey of Health, Ageing and Retirement in Europe (SHARE). Moreover, we examined whether these associations varied by sex. We hypothesised that greater pain intensity compared to no pain would be associated with a higher risk of significant depressive symptoms, in the overall study population and in both sexes.

## Methods

### Study design and population

SHARE is an ongoing, longitudinal, biennial panel survey of ageing processes in individuals in European countries and Israel, as previously described [30–32]. In brief, nationally-representative samples from European countries and Israel were drawn based on probability household samples; eligible households had at least one non-institutionalised member ≥ 50 years who spoke the official language of the country and was not living abroad at the time of the survey. Eligible participants were individuals ≥ 50 years and their partners, irrespective of age. They were followed over time and refreshment samples of new individuals were enrolled in new waves to compensate for dropout. Participants were interviewed by trained interviewers using standardised computer-assisted personal interviews, in either the participant’s household or nursing home or unknown setting. Participants who gave consent had a face-to-face “main interview”. If participants were deceased, their proxy could take part in an “End of Life interview”. If participants could not be contacted, interviews were “missing”. The main interview included several sections (e.g., demographics, physical health, mental health); for each section, participants were direct respondents or respondents by proxy.

In the present study, we used data from Wave 5 (baseline) and Wave 6 (follow-up), which were collected in 2013 and 2015, respectively [31, 32]. These Waves were chosen due to the availability of data of interest.

At baseline, 66,065 participants had a main interview. We excluded those < 50 years or of unknown age (n = 1178), those from the Netherlands (n = 4116) (as the Netherlands participated in Wave 5 but not 6) or in a nursing home or unknown setting (n = 654), those who were not direct respondents on pain (n = 2833), those with a diagnosis of cognitive decline or unknown (n = 511) and those with missing data on either pain (n = 62) or depressive symptoms as measured using the self-assessment questionnaire EURO-D [33–35] (n = 717) or other co-variates (n = 1985). Furthermore, we excluded 13,615 participants with significant depressive symptoms at baseline, as defined by a EURO-D score ≥ 4, which is indicative of major depression, based on clinical criteria [33–36]. We further excluded participants with a diagnosis of affective or emotional disorder (n = 1061) and those taking drugs for anxiety or depression (n = 738) or sleep (n = 1349). Therefore, we had a sample of 37,242 participants with no indication of depression at baseline. At follow-up, we further excluded 8727 participants with no main interview (at Wave 6), or with a new diagnosis of cognitive decline or unknown (at Wave 6), or missing data on depressive symptoms (at Wave 6). Supplementary Table 1 details differences in baseline characteristics between participants who were lost to follow-up between Wave 5 and 6 and those participants who were included in our study (retention rate 28,515/37,242 = 76.6%). In particular, those who were lost to follow-up between Wave 5 and 6 were more likely to be men, and to report moderate or severe pain, fair or poor self-rated health, mobility and functional impairment at baseline, compared to those who were included in our study.

**Fig. 1:**
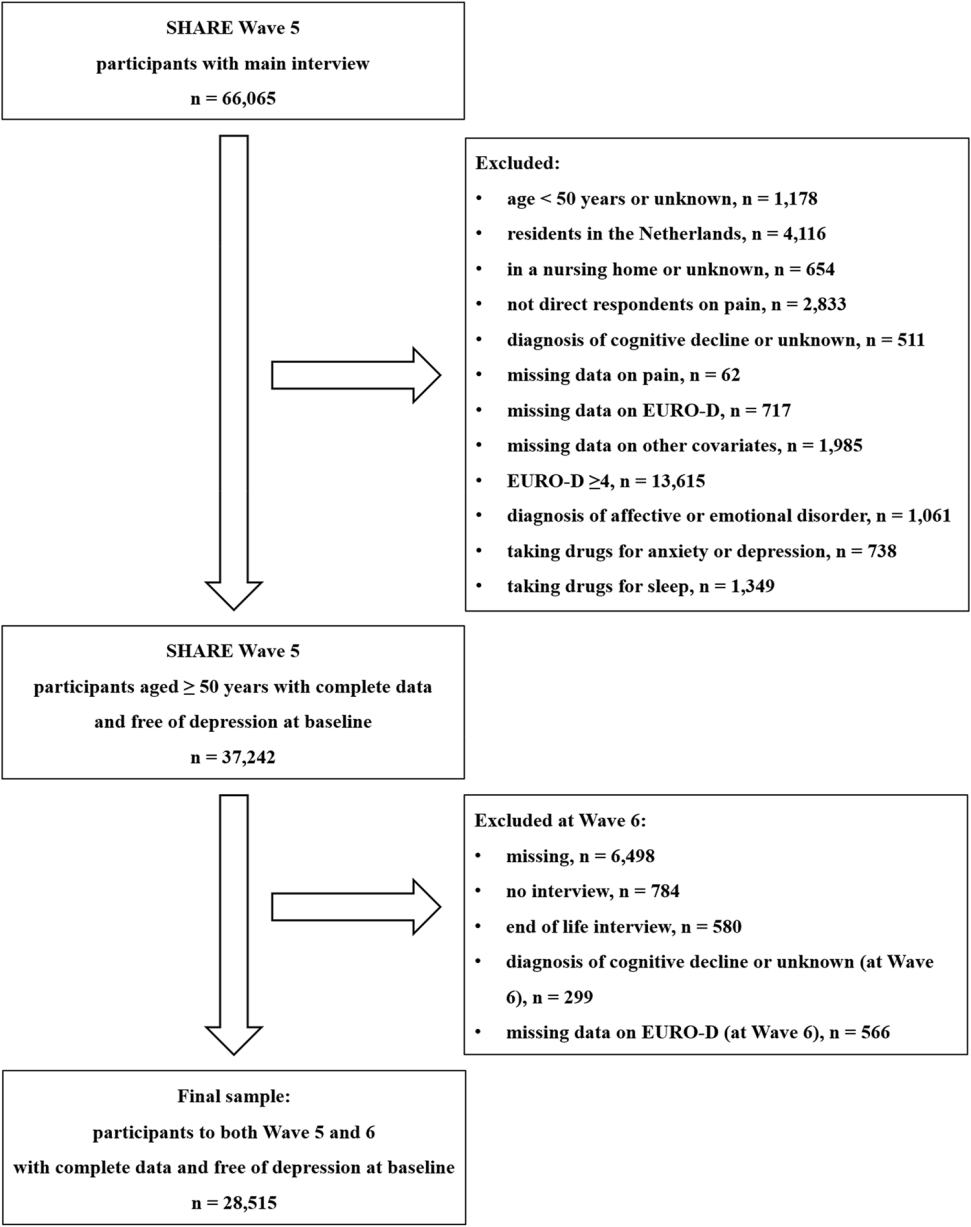
Flow-chart of study inclusion criteria. *SHARE* Survey of Health, Ageing and Retirement in Europe, *n* number. Please, note that this paper is based on data on SHARE Wave 5 and 6 Release version: 8.0.0 (10th February 2022). The numbers of participants slightly differ from those reported in a previous paper of ours that used data of Release version: 7.1.0 (26th June 2020). Every time a new SHARE wave is released, all previous waves get updated. Sometimes participants may decide to drop out of the survey and request to have all information deleted. Other observations may be deleted due to implausible or inconsistent reporting

Therefore, this study included 28,515 community-dwelling participants ≥ 50 years, free of depression at baseline, with follow-up at two years and complete data of interest (Fig. 1). They were residents from 14 countries: Austria, Belgium, Czech Republic, Denmark, Estonia, France, Germany, Israel, Italy, Luxembourg, Slovenia, Spain, Sweden and Switzerland.

We excluded those with a diagnosis of cognitive impairment or unknown because the risk factors for depression differ in adults with and without cognitive impairment [37] and because cognitive impairment may affect the validity of self-reported questionnaires for depression such as EURO-D [38, 39]. We excluded those with significant depressive symptoms or a history of depression or taking psychotropic medications at baseline to rule out reverse causation in the association between pain intensity and depressive symptoms.

### Demographic and clinical characteristics

At baseline, socio-demographic and clinical characteristics were recorded. Age, sex and country were collected. Educational level was classified based on the International Standard Classification of Education (ISCED)-97 [40], as follows: none or pre-primary; primary; lower secondary; upper secondary; post-secondary non-tertiary; first stage of tertiary; second stage of tertiary education; still in school and other. We merged the categories “still in school” and “other”.

We dichotomised self-rated health as good (“excellent” or “very good” or “good”) versus fair or poor (“fair” or “poor”). Body mass index (BMI, kg/m2) was computed from self-reported height and weight. BMI categories were defined as follows: underweight (BMI < 18.5 kg/m^2^), normal weight (BMI ≥ 18.5 and < 25 kg/m^2^), overweight (BMI ≥ 25 and < 30 kg/m^2^)) and with obesity (BMI ≥ 30 kg/m^2^) [41].

Co-morbidities were ascertained by asking the participants: “Has a doctor ever told you that you had/ do you currently have any of the conditions on this card?” and a list was shown. Based on previous literature on risk factors for depression [12], we selected these cardiovascular risk factors and co-morbidities: heart attack, hypertension, high cholesterol, stroke, diabetes mellitus (any type), chronic lung disease, cancer, Parkinson’s, hip fracture, other fractures, rheumatoid arthritis, osteoarthritis/other rheumatism.

Medications were inquired by asking: “Do you currently take drugs, at least once a week, for problems mentioned on this card?” and a list was shown. Based on previous literature [12], we selected these medications: drugs for joint pain, drugs for other pain and drugs for suppressing inflammation (only glucocorticoids or steroids).

Participants were classified as living alone versus not, based on the number of people living in their household.

Participants were asked: “How often do you engage in vigorous physical activity, such as sports, heavy housework, or a job that involves physical labour?” and “How often do you engage in activities that require a moderate level of energy such as gardening, cleaning the car, or doing a walk?”. Physical inactivity was defined as neither vigorous nor moderate physical activity.

Loneliness was assessed using a short three-item version [42] of the Revised UCLA Loneliness scale [43]. Participants were asked: “How much of the time do you feel you lack companionship?”, “How much of the time do you feel left out?” and “How much of the time do you feel isolated from others?”. Possible options were: “Often”, “Some of the time” and “Hardly ever or never”, which were scored 3, 2 and 1 point, respectively. The sum score ranged from 3 (not lonely) to 9 (very lonely).

Mobility impairment was defined by self-reported difficulties with three or more of ten tasks, as detailed in the SHARE questionnaire. Impairment in Activities of Daily Living (ADL impairment) was defined by self-reported difficulties with one or more of six ADLs (dressing, walking across a room, bathing, eating, getting in or out of bed, using toilet). Impairment in Instrumental ADL (IADL impairment) was defined by self-reported difficulties with one or more of seven IADLs (using a map, preparing a hot meal, shopping, making telephone calls, taking medications, doing work around the house or garden, managing money).

### Pain

At baseline, participants were asked: “Are you troubled with pain?”, options were “yes” or “no”. Participants reporting pain were then asked to describe the intensity of pain as “mild”, “moderate” or “severe”. Participants were divided into these categories: no pain, mild pain, moderate pain or severe pain. A similar classification has been used in the English Longitudinal Study of Ageing (ELSA) [18] and in the Health and Retirement Study (HRS) [26]. SHARE is harmonized with these studies [30].

### Depressive symptoms

Depressive symptoms were assessed using the EURO-D scale, a self-assessment questionnaire that was validated in adults across European and non-European countries [33–35]. It comprises 12 items (depressed mood, pessimism, “would rather be dead” feelings, guilt, poor sleep, lack of interest, irritability, poor appetite, fatigue, lack of concentration, lack of enjoyment, and tearfulness), which are scored 0 (symptom not present) or 1 (symptom present), with the reference period being the last month [33, 34]. The total sum score ranges between 0 and 12, with higher values indicating higher burden of depressive symptoms [33, 34]. As in previous studies [14, 15, 33–35], we used the cut-off of ≥ 4 points to define the presence of significant depressive symptoms. This cut-off is indicative of major depression as defined by DSM-IV criteria [33–36].

### Statistical analyses

We reported the baseline characteristics of our study population by: (1) pain intensity and (2) sex. We tested for differences in baseline characteristics using Pearson’s chi-square test for categorical variables and student’s t test or analysis of variance (ANOVA), as appropriate, for continuous variables.

Logistic regression models were used to assess the longitudinal association between pain intensity at baseline and the risk of significant depressive symptoms at follow-up. The category “no pain” was set as the reference. We adjusted all our analyses according to three Models. In Model 1, we adjusted our analyses for age (continuous variable) and sex. In Model 2, we further adjusted for educational level, self-rated health, BMI category, co-morbidities, medications, living alone and country. In Model 3, we adjusted for all co-variates of Model 2 and physical inactivity, mobility impairment, ADL impairment, IADL impairment and loneliness. In the total sample of participants, we further adjusted for the number of depressive symptoms (EURO-D score) at baseline, to account for subclinical depression (Model 4).

We computed interaction terms as sex (as coded as follows: 1 = male; 2 = female) by pain intensity (as coded as follows: 1 = no pain; 2 = mild pain; 3 = moderate pain; 4 = severe pain), to test whether sex may modify the associations between pain intensity at baseline and the risk of significant depressive symptoms at follow-up.

Furthermore, we performed sub-group analyses by stratifying the participants into two age groups: participants aged 50–64 years versus those aged ≥ 65 years. To explore the influence of mobility and functional impairments and loneliness on the pain intensity—depressive symptoms association, we performed sensitivity analyses by excluding: (1) participants with mobility or ADL or IADL impairment and (2) participants with one or more symptoms of loneliness, respectively. To further investigate the role of subclinical depression at baseline, we re-ran the analyses by restricting the sample to participants with no or only one depressive symptom (i.e. EURO-D score 0–1) at baseline.

To explore potential reverse causation, we investigated whether higher number of depressive symptoms at baseline (determinant) may be associated with a higher risk of worsening pain intensity from baseline to follow-up (binary outcome), using binary regression models.

All analyses were performed using SPSS software (version 25). We considered a p value ≤ 0.10 to be statistically significant for interaction and a p value ≤ 0.05 for all other analyses.

### Ethics

SHARE obtained ethical approval by the University of Mannheim, during Waves 1 to 4, and by the Ethics Council of the Max Planck Society for Wave 4 and the continuation of the project [30–32]. All participants signed an informed consent [30–32].

## Results

### Characteristics at baseline

Baseline interviews within SHARE were conducted from January to December 2013. Our study population included 28,515 community-dwelling participants, without diagnosis of cognitive decline, or any affective or emotional disorders, or use of drugs for sleep, or anxiety or depression or significant depressive symptoms at baseline. Among the participants, 14,360 (50.4%) were women, 4810 (16.9%) reported less than secondary education, and 6922 (24.3%) fair or poor self-rated health. Age ranged from 50 to 99 years; mean age was 65.4 (standard deviation 9.0) years. Overall, 7941 (27.8%) participants reported one or more symptoms of loneliness, while 4799 (16.8%) participants reported mobility and or ADL and or IADL impairment.

At baseline, 19,028 (66.7%) participants reported no pain, 2803 (9.8%) mild pain, 5253 (18.4%) moderate pain and 1431 (5.0%) severe pain (Table 1). These proportions varied by country, in the overall study population and in both sexes (Supplementary Tables 2 and 3) Participants with severe pain were older compared to those without pain (Table 1). The proportion of women, of participants with less than secondary education, and of participants reporting fair or poor health, obesity, most co-morbidities (except stroke, cancer and Parkinson’s disease), use of medications for pain, living alone, physical inactivity, loneliness, mobility and functional impairment were higher with increasing pain intensity, being lowest in those with no pain, intermediate in those with mild and moderate pain and highest in those with severe pain (Table 1). The proportion of participants reporting no or only one depressive symptom at baseline ranged from 67.3% to 40.1% among those with no pain and those with severe pain, respectively (Table 1).

**Table 1:** Characteristics of study population at baseline stratified by pain intensity

	All (n = 28,515)	No pain (n = 19,028)	Mild pain (n = 2803)	Moderate pain (n = 5253)	Severe pain (n = 1431)	p value
Age (years), mean (SD)	65.4 (9.0)	64.9 (8.8)	65.5 (8.7)	66.7 (9.2)	66.8 (9.6)	< 0.001
Women, n (%)	14,360 (50.4)	9067 (47.7)	1451 (51.8)	3005 (57.2)	837 (58.5)	< 0.001
Educational level, n (%)						
None or pre-primary	861 (3.0)	500 (2.6)	84 (3.0)	209 (4.0)	68 (4.8)	< 0.001
Primary	3949 (13.8)	2338 (12.3)	415 (14.8)	918 (17.5)	278 (19.4)	
Lower secondary	4515 (15.8)	2805 (14.7)	473 (16.9)	962 (18.3)	275 (19.2)	
Upper secondary	10,087 (35.4)	6782 (35.6)	941 (33.6)	1870 (35.6)	494 (34.5)	
Post-secondary non-tertiary	1471 (5.2)	1039 (5.5)	144 (5.1)	236 (4.5)	52 (3.6)	
First stage of tertiary	7264 (25.5)	5317 (27.9)	698 (24.9)	997 (19.0)	252 (17.6)	
Second stage of tertiary	285 (1.0)	191 (1.0)	38 (1.4)	46 (0.9)	10 (0.7)	
Still in school or other	83 (0.3)	56 (0.3)	10 (0.4)	15 (0.3)	2 (0.1)	
Fair or poor self-rated health, n (%)	6922 (24.3)	2914 (15.3)	713 (25.4)	2372 (45.2)	923 (64.5)	< 0.001
BMI category, n (%)						
Underweight	257 (0.9)	183 (1.0)	20 (0.7)	38 (0.7)	16 (1.1)	< 0.001
Normal weight	10,445 (36.6)	7537 (39.6)	964 (34.4)	1557 (29.6)	387 (27.0)	
Overweight	12,141 (42.6)	8121(42.7)	1236 (44.1)	2236 (42.6)	548 (38.3)	
With obesity	5672 (19.9)	3187 (16.7)	583 (20.8)	1422 (27.1)	480 (33.5)	
Co-morbidities, n (%)						
Heart attack	2507 (8.8)	1411 (7.4)	242 (8.6)	620 (11.8)	234 (16.4)	< 0.001
Hypertension	10,633 (37.3)	6422 (33.8)	1109 (39.6)	2428 (46.2)	674 (47.1)	< 0.001
High cholesterol	6038 (21.2)	3692 (19.4)	650 (23.2)	1308 (24.9)	388 (27.1)	< 0.001
Stroke	651 (2.3)	371 (1.9)	53 (1.9)	166 (3.2)	61 (4.3)	< 0.001
Diabetes	3069 (10.8)	1758 (9.2)	318 (11.3)	749 (14.3)	244 (17.1)	< 0.001
Chronic lung disease	1145 (4.0)	624 (3.3)	94 (3.4)	312 (5.9)	115 (8.0)	< 0.001
Cancer	1270 (4.5)	804 (4.2)	110 (3.9)	257 (4.9)	99 (6.9)	< 0.001
Parkinson’s disease	104 (0.4)	51 (0.3)	7 (0.2)	30 (0.6)	16 (1.1)	< 0.001
Hip fracture	345 (1.2)	173 (0.9)	43 (1.5)	98 (1.9)	31 (2.2)	< 0.001
Other fracture	1410 (4.9)	736 (3.9)	166 (5.9)	366 (7.0)	142 (9.9)	< 0.001
Rheumatoid arthritis	1809 (6.3)	488 (2.6)	270 (9.6)	759 (14.4)	292 (20.4)	< 0.001
Osteoarthritis/other rh	4113 (14.4)	1360 (7.1)	591 (21.1)	1625 (30.9)	537 (37.5)	< 0.001
Drugs, n (%)						
For joint pain	3096 (10.9)	536 (2.8)	409 (14.6)	1543 (29.4)	608 (42.5)	< 0.001
For other pain	2133 (7.5)	470 (2.5)	298 (10.6)	952 (18.1)	413 (28.9)	< 0.001
For inflammation*	634 (2.2)	188 (1.0)	86 (3.1)	263 (5.0)	97 (6.8)	< 0.001
Lives alone, n (%)	5290 (18.6)	3415 (17.9)	515 (18.4)	1043 (19.9)	317 (22.2)	< 0.001
Physical inactivity, n (%)	1536 (5.4)	715 (3.8)	129 (4.6)	470 (8.9)	222 (15.5)	< 0.001
Loneliness score, n (%)						
3 (not lonely)	20,574 (72.2)	14,203 (74.6)	1927 (68.7)	3499 (66.6)	945 (66.0)	< 0.001
4	4637 (16.3)	2944 (15.5)	500 (17.8)	943 (18.0)	250 (17.5)	
5	1885 (6.6)	1122 (5.9)	236 (8.4)	415 (7.9)	112 (7.8)	
6	999 (3.5)	541 (2.8)	102 (3.6)	280 (5.3)	76 (5.3)	
7	277 (1.0)	146 (0.8)	28 (1.0)	76 (1.4)	27 (1.9)	
8	76 (0.3)	43 (0.2)	4 (0.1)	20 (0.4)	9 (0.6)	
9 (very lonely)	67 (0.2)	29 (0.2)	6 (0.2)	20 (0.4)	12 (0.8)	
Mobility impairment	3616 (12.7)	986 (5.2)	363 (13.0)	1559 (29.7)	708 (49.5)	< 0.001
ADL impairment	1296 (4.5)	405 (2.1)	106 (3.8)	517 (9.8)	268 (18.7)	< 0.001
IADL impairment	2068 (7.3)	763 (4.0)	180 (6.4)	785 (14.9)	340 (23.8)	< 0.001
EURO-D score, n (%)						
0	9122 (32.0)	7049 (37.0)	769 (27.4)	1107 (21.1)	197 (13.8)	< 0.001
1	8488 (29.8)	5768 (30.3)	836 (29.8)	1507 (28.7)	377 (26.3)	
2	6320 (22.2)	3766 (19.8)	733 (26.2)	1415 (26.9)	406 (28.4)	
3	4585 (16.1)	2445 (12.8)	465 (16.6)	1224 (23.3)	451 (31.5)	

*n* number, *SD* standard deviation, *BMI* body mass index, *other rh.* other rheumatism, *ADL* activities of daily living, *IADL* instrumental activities of daily living

*Drugs for inflammation drugs for suppressing inflammation (only glucocorticoids or steroids). p values were computed by Pearson’s chi-square for categorical variables, and by ANOVA for age (continuous variable)

Women were more likely to report pain and higher pain intensity than men (Supplementary Table 4). Most socio-demographic and clinical characteristics varied by sex (Supplementary Table 4). A higher proportion of women reported rheumatoid arthritis, osteoarthritis/other rheumatism, use of medications for pain, physical inactivity, living alone, loneliness, mobility and IADL impairment compared to men (Supplementary Table 4). A higher proportion of men reported fair or poor self-rated health, overweight, and a physician diagnosis of heart attack, hypertension, high cholesterol, stroke, diabetes, chronic lung disease and Parkinson’s disease, compared to women (Supplementary Table 4). A higher proportion of men reported no or only one depressive symptom at baseline compared to women (Supplementary Table 4).

### Depressive symptoms at follow-up

Follow-up interviews were conducted from January to November 2015. Mean time interval between baseline and follow-up interview was 23.4 (SD 3.4) months, with median 23 months (interquartile range 21–25 months). Overall, 3868 (13.6%) participants reported significant depressive symptoms at follow-up. In particular, 1451 (10.3%) men and 2417 (16.8%) women reported significant depressive symptoms, with variation across countries (Supplementary Table 5). Women reported significant depressive symptoms more frequently than men (Table 2, p < 0.001).

**Table 2:** Depressive symptoms at follow-up, by sex

	All (n = 28,515)	Men (n = 14,155)	Women (n = 14,360)	p value
Significant depressive symptoms, (EURO-D score ≥ 4), n (%)	3868 (13.6)	1451 (10.3)	2417 (16.8)	< 0.001
EURO-D items, n (%)				
Feeling sad or depressed	8255 (28.9)	3252 (23.0)	5003 (34.8)	< 0.001
Pessimism	3153 (11.1)	1600 (11.3)	1553 (10.8)	0.188
Would rather be dead	755 (2.6)	340 (2.4)	415 (2.9)	0.010
Guilt	1421 (5.0)	546 (3.9)	875 (6.1)	< 0.001
Poor sleep	7447 (26.1)	2954 (20.9)	4493 (31.3)	< 0.001
Lack of interest	1305 (4.6)	643 (4.5)	662 (4.6)	0.785
Irritability	6107 (21.4)	3083 (21.8)	3024 (21.1)	0.137
Poor appetite	1542 (5.4)	660 (4.7)	882 (6.1)	< 0.001
Fatigue	7750 (27.2)	3491 (24.7)	4259 (29.7)	< 0.001
Lack of concentration	2850 (10.0)	1482 (10.5)	1368 (9.5)	0.008
Lack of enjoyment	2265 (7.9)	1156 (8.2)	1109 (7.7)	0.166
Tearfulness	4485 (15.7)	1193 (8.4)	3292 (22.9)	< 0.001

p values were computed by Pearson’s chi-square for categorical variables

*n* number

The most frequently reported depressive symptoms were feeling sad or depressed, poor sleep and fatigue among women; among men, they were fatigue, feeling sad or depressed and irritability (Table 2). The least reported symptom was “would rather be dead” feelings in both men and women (Table 2).

The distribution of specific depressive symptoms varied by sex (Table 2). In detail, a higher proportion of women than men reported feeling sad or depressed, “would rather be dead” feelings, guilt, poor sleep, poor appetite, fatigue and tearfulness (Table 2). In contrast, a higher proportion of men than women reported lack of concentration (Table 2). Similar proportions of men and women reported pessimism, lack of interest, irritability and lack of enjoyment (Table 2).

The proportion of participants reporting significant depressive symptoms as well as most specific depressive symptoms (except guilt and lack of enjoyment) at follow-up was higher with greater pain intensity at baseline (Table 3). In detail, 2030 (10.7%) participants with no baseline pain reported significant depressive symptoms at follow-up, compared with 421 (15.0%), 1057 (20.1%) and 360 (25.2%) participants with mild, moderate and severe pain, respectively (Table 3).

**Table 3:** Depressive symptoms at follow-up by pain intensity at baseline

	All (n = 28,515)	No pain (n = 19,028)	Mild pain (n = 2803)	Moderate pain (n = 5253)	Severe pain (n = 1431)	p value
Significant depressive symptoms, (EURO-D score ≥ 4), n (%)	3868 (13.6)	2030 (10.7)	421 (15.0)	1057 (20.1)	360 (25.2)	< 0.001
EURO-D items, n (%)						
Feeling sad or depressed	8255 (28.9)	4932 (25.9)	907 (32.4)	1865 (35.5)	551 (38.5)	< 0.001
Pessimism	3153 (11.1)	1939 (10.2)	324 (11.6)	657 (12.5)	233 (16.3)	< 0.001
Would rather be dead	755 (2.6)	421 (2.2)	81 (2.9)	174 (3.3)	79 (5.5)	< 0.001
Guilt	1421 (5.0)	882 (4.6)	171 (6.1)	301 (5.7)	67 (4.7)	< 0.001
Poor sleep	7447 (26.1)	4385 (23.0)	824 (29.4)	1711 (32.6)	527 (36.8)	< 0.001
Lack of interest	1305 (4.6)	742 (3.9)	140 (5.0)	316 (6.0)	107 (7.5)	< 0.001
Irritability	6107 (21.4)	3683 (19.4)	700 (25.0)	1338 (25.5)	386 (27.0)	< 0.001
Appetite	1542 (5.4)	901 (4.7)	136 (4.9)	353 (6.7)	152 (10.6)	< 0.001
Fatigue	7750 (27.2)	4241 (22.3)	869 (31.0)	1978 (37.7)	662 (46.3)	< 0.001
Lack of concentration	2850 (10.0)	1668 (8.8)	316 (11.3)	665 (12.7)	201 (14.0)	< 0.001
Lack of enjoyment	2265 (7.9)	1411 (7.4)	191 (6.8)	519 (9.9)	144 (10.1)	< 0.001
Tearfulness	4485 (15.7)	2705 (14.2)	492 (17.6)	995 (18.9)	293 (20.5)	< 0.001

p values were computed by Pearson’s chi-square for categorical variables

*n* number

### Pain intensity at baseline and risk of significant depressive symptoms at follow-up

In age- and sex-adjusted analyses, participants with mild, moderate, and severe baseline pain had an increased risk of significant depressive symptoms of 1.44 (1.28–1.61), 1.96 (1.80–2.13) and 2.60 (2.28–2.96), respectively, compared to those without pain (Model 1, Table 4). These associations remained significant after further adjusting for educational level, self-rated health, BMI category, co-morbidities, medications, living alone and country (Model 2, Table 4).

**Table 4:** Longitudinal association between pain intensity at baseline and significant depressive symptoms at follow-up, by sex

	All (n = 28,515)		Men (n = 14,155)		Women (n = 14,360)		P for interaction
	OR [95% CI]	p value	OR [95% CI]	p value	OR [95% CI]	p value	
Model 1							
No pain	1 (ref)		1 (ref)		1 (ref)		0.057
Mild pain	1.44 [1.28; 1.61]	< 0.001	1.51 [1.26; 1.81]	< 0.001	1.39 [1.20; 1.61]	< 0.001	
Moderate pain	1.96 [1.80; 2.13]	< 0.001	2.15 [1.88; 2.45]	< 0.001	1.85 [1.67; 2.06]	< 0.001	
Severe pain	2.60 [2.28; 2.96]	< 0.001	2.85 [2.30; 3.52]	< 0.001	2.46 [2.09; 2.90]	< 0.001	
Model 2							
No pain	1 (ref)		1 (ref)		1 (ref)		0.042
Mild pain	1.22 [1.08; 1.37]	0.001	1.32 [1.10; 1.60]	0.004	1.16 [0.99; 1.35]	0.066	
Moderate pain	1.42 [1.29; 1.57]	< 0.001	1.66 [1.42; 1.93]	< 0.001	1.30 [1.15; 1.47]	< 0.001	
Severe pain	1.58 [1.36; 1.84]	< 0.001	1.88 [1.47; 2.39]	< 0.001	1.43 [1.18; 1.73]	< 0.001	
Model 3							
No pain	1 (ref)		1 (ref)		1 (ref)		0.033
Mild pain	1.20 [1.06; 1.35]	0.003	1.29 [1.07; 1.56]	0.008	1.13 [0.97; 1.32]	0.119	
Moderate pain	1.32 [1.20; 1.46]	< 0.001	1.52 [1.30; 1.78]	< 0.001	1.21 [1.06; 1.37]	0.003	
Severe pain	1.39 [1.19; 1.63]	< 0.001	1.59 [1.24; 2.05]	< 0.001	1.28 [1.05; 1.56]	0.013	
Model 4							
No pain	1 (ref)		1 (ref)		1 (ref)		0.048
Mild pain	1.14 [1.01; 1.28]	0.037	1.23 [1.01; 1.48]	0.037	1.08 [0.92; 1.26]	0.362	
Moderate pain	1.22 [1.10; 1.34]	< 0.001	1.39 [1.19; 1.64]	< 0.001	1.11 [0.98; 1.26]	0.098	
Severe pain	1.22 [1.04; 1.42]	0.014	1.34 [1.04; 1.73]	0.023	1.13 [0.93; 1.38]	0.216	

Odds ratios and 95% confidence intervals were calculated by binary logistic regression. Model 1: adjusted for age and sex. Model 2: Model 1 + education, self-rated health, body mass index (BMI) category, heart attack, hypertension, high cholesterol, stroke, diabetes, chronic lung disease, cancer, Parkinson’s, hip fracture, other fractures, rheumatoid arthritis, osteoarthritis or other rheumatism, drugs for joint pain, drugs for other pain, drugs for suppressing inflammation (only glucocorticoids or steroids), living alone, country. Model 3: Model 2 + physical inactivity, mobility impairment, Activities of Daily Living (ADL) impairment, Instrumental ADL (IADL) impairment, loneliness. Model 4: Model 3 + number of depressive symptoms at baseline (EURO-D score at baseline, continuous variable)

After adjustment for Model 3, mild, moderate, and severe pain, versus no pain, were longitudinally associated with an increased likelihood of significant depressive symptoms of 1.20 (1.06–1.35), 1.32 (1.20–1.46) and 1.39 (1.19–1.63), respectively (Table 4).

After adjustment for Model 3, men with mild, moderate, and severe pain at baseline, respectively, had a higher risk of significant depressive symptoms at follow-up of 1.29 (1.07–1.56), 1.52 (1.30–1.78) and 1.59 (1.24–2.05), compared to those without pain (Table 4). After adjustment for Model 3, women with moderate and severe pain at baseline, respectively, had a higher risk of significant depressive symptoms at follow-up of 1.21 (1.06–1.37) and 1.28 (1.05–1.56), compared to those without pain (Table 4). Among women, no longitudinal association was found between mild pain at baseline and significant depressive symptoms at follow-up (Model 3, Table 4). The longitudinal association between pain intensity and significant depressive symptoms at follow-up varied by sex (p for interaction = 0.033, Model 3, Table 4).

After further adjusting for EURO-D score at baseline, the longitudinal association between greater pain intensity at baseline and higher risk of significant depressive symptoms at follow-up remained consistent in the overall study population and in men (Model 4, Table 4).

### Sensitivity analyses

After adjustment for Model 3, the association between mild, moderate and severe pain at baseline, compared to no pain, and higher risk of significant depressive symptoms at follow-up remained consistent among participants aged 50–64 years (Table 5). Among participants ≥ 65 years, moderate and severe pain at baseline were associated with higher risk of significant depressive symptoms at follow-up, compared to no pain, while mild pain was not (Model 3, Table 5).

**Table 5:** Longitudinal association between pain intensity at baseline and significant depressive symptoms at follow-up, by age categories

	Aged 50 to 64 years (n = 14,070)		Aged ≥ 65 years (n = 14,445)	
	OR [95% CI]	p value	OR [95% CI]	p value
Model 1				
No pain	1 (ref)		1 (ref)	
Mild pain	1.59 [1.35; 1.87]	< 0.001	1.32 [1.13; 1.55]	0.001
Moderate pain	1.96 [1.73; 2.22]	< 0.001	1.96 [1.75; 2.18]	< 0.001
Severe pain	2.30 [1.87; 2.82]	< 0.001	2.78 [2.35; 3.29]	< 0.001
Model 2				
No pain	1 (ref)		1 (ref)	
Mild pain	1.36 [1.15; 1.61]	< 0.001	1.08 [0.92; 1.28]	0.354
Moderate pain	1.46 [1.27; 1.69]	< 0.001	1.38 [1.21; 1.57]	< 0.001
Severe pain	1.44 [1.14; 1.83]	0.002	1.65 [1.35; 2.00]	< 0.001
Model 3				
No pain	1 (ref)		1 (ref)	
Mild pain	1.31 [1.10; 1.55]	0.002	1.08 [0.91; 1.28]	0.386
Moderate pain	1.37 [1.18; 1.59]	< 0.001	1.28 [1.12; 1.46]	< 0.001
Severe pain	1.30 [1.02; 1.66]	0.034	1.45 [1.19; 1.78]	< 0.001

Odds ratios and 95% confidence intervals were calculated by binary logistic regression. Model 1: adjusted for age and sex. Model 2: Model 1 + education, self-rated health, body mass index (BMI) category, heart attack, hypertension, high cholesterol, stroke, diabetes, chronic lung disease, cancer, Parkinson’s, hip fracture, other fractures, rheumatoid arthritis, osteoarthritis or other rheumatism, drugs for joint pain, drugs for other pain, drugs for suppressing inflammation (only glucocorticoids or steroids), living alone, country. Model 3: Model 2 + physical inactivity, mobility impairment, Activities of Daily Living (ADL) impairment, Instrumental ADL (IADL) impairment, loneliness

The longitudinal associations between higher intensity of pain at baseline and higher risk of significant depressive symptom at follow-up remained significant when restricting the analyses to: (1) the sample of participants without mobility or ADL or IADL impairment (n = 23,716); and (2) the sample of participants with no symptom of loneliness at baseline (n = 20,574) (Model 3, Supplementary Table 6 and 7, respectively).

When restricting the analyses to participants with EURO-D score 0–1 at baseline (n = 17,610), the longitudinal association between higher pain intensity at baseline and greater risk of significant depressive symptoms at follow-up was significant among men, but not among women (Model 3, Supplementary Table 8).

Out of 28,502 participants, 6002 (21.1%) had worsening pain intensity from baseline to follow-up (Supplementary Table 9). A higher number of depressive symptoms at baseline was longitudinally associated with a higher risk of worsening pain intensity from baseline to follow-up (Supplementary Table 10).

## Discussion

In this large, cross-national, prospective study, greater pain intensity at baseline was longitudinally associated with higher risk of significant depressive symptoms at follow-up, in community-dwelling adults with no significant depressive symptoms or history of depression or use of psychotropic medications at baseline. These associations were only partly explained by socio-demographic and clinical factors, including educational level, self-rated health, co-morbidities, pain medications, living alone, physical inactivity, loneliness, mobility and functional impairments. These associations between baseline pain intensity and risk of significant depressive symptoms at follow-up were more pronounced in men compared to women.

Our study adds to the literature on pain and depressive symptoms in multiple ways. A previous study using data from SHARE showed the association between presence of pain and depressive symptoms, but did not investigate the association between pain intensity and onset of significant depressive symptoms [44].

As in previous literature, women were more likely to report pain [29] and develop depression than men [13]. Pain and depression co-exist more frequently in women. However, the novelty of our study is to show the association between greater pain intensity at baseline and greater risk of onset of depressive symptoms at follow-up and that this association was more accentuated in men compared to women. A previous population-based study showed the associations between four aspects of the pain experience (presence, frequency of episodes, duration and number of pain locations) and depressive symptoms among 70-years-old Swedish adults, and also found that these associations were more pronounced among men than women [45]. In contrast, a pooled analysis of data from ELSA and the China Health Retirement Longitudinal Study showed a longitudinal association between baseline pain intensity and incident depressive symptoms, in adults without depressive symptoms at baseline, which did not differ by sex [18]. Our study adds to previous literature by exploring the pain-depression association in a large diverse cohort of adults from European countries and Israel, depicting the cross-national variation in depressive symptoms. Moreover, we investigated the role of physical inactivity, loneliness, mobility and functional impairments (potential mediators) as well as subclinical depression (potential reverse causation).

Pain may be linked to the onset of depressive symptoms through various pathways.

First, pain and depressive symptoms may share both protective and risk factors. Higher educational level may protect against both pain and depression, as people with higher educational level may have healthier lifestyles and better access to medical care. Pain may be a symptom of underlying co-morbidities, such as stroke or rheumatoid arthritis, which are known risk factors for depression. Obesity may be a common risk factor for both pain and depression [46–48]. Obesity has been linked with lower pain threshold and tolerance. Moreover, it leads to higher mechanical overload on weight-bearing joints, greater cartilage damage and thus joint pain in the lower extremities [47]. Obesity has been associated with chronic low-grade inflammation, which can result in hyperalgesia [47]. On the other side, overweight and obesity have been longitudinally associated with onset of depression in adults [48]. Obesity may lead to depression through various biological pathways, including activation of inflammation and dysregulation of the hypothalamic–pituitary–adrenal (HPA) axis. It may lead to depression because of sociocultural factors; if thinness is considered a beauty ideal, then obesity may trigger body dissatisfaction, lower self-esteem and then depression [48]. However, in our study, the association between pain and depressive symptoms remained significant after adjusting for educational level, co-morbidities, and BMI categories.

Second, pain may be causally linked to depression through various mechanisms. Pain may lead to physical inactivity [19], which is a known risk factor for depression in adults [15]. Consistent with previous literature [19], our study showed that participants with greater pain intensity were more likely to be physically inactive. Yet, our findings remained consistent after adjusting for physical inactivity.

Moreover, pain may contribute to mobility and functional limitation. In the Women’s Health and Aging Study, about one third of community-dwelling older women reported musculoskeletal pain symptoms as the main cause of their mobility and or functional impairment [49]. In the HRS, community-dwelling participants aged ≥ 50 years with pain were similar in terms of their degree of functional limitation to participants without pain who were 2 to 3 decades older [20]. Both mobility and functional impairment are known risk factors for depression [12]. However, our findings remained consistent after adjusting for mobility and functional impairment as well as in sensitivity analyses in participants without mobility or functional impairment.

Furthermore, pain may lead to social isolation and loneliness, which favour the onset of depressive symptoms. In ELSA, baseline moderate to severe pain was associated with loneliness four years later [23]. Similarly, pain was associated with loneliness in older adults in their last years of life [22]. However, our findings were consistent after adjusting for living alone and symptoms of loneliness and also after excluding participants with one or more symptoms of loneliness.

From a biological perspective, pain may be causally linked to depression, through subclinical chronic inflammation and activation of the HPA axis. Yet, our associations remained consistent after adjustment for many co-morbidities that are associated with subclinical chronic inflammation.

Assuming that pain may be causally linked to onset of depression, our findings would imply that men may be more vulnerable to depression in relation to pain compared to women. Further studies should explore how the subjective experience of pain may be mediated by sex and gender.

Further studies should explore the bidirectional association between pain and depression, and whether depression, including subclinical depression, may be causally linked to changes in pain intensity. In our study, participants with a higher number of depressive symptoms at baseline had a greater risk of worsening pain intensity at follow-up. A major strength of our population-based study is the longitudinal design and the exclusion of participants with depression—as defined by significant depressive symptoms, diagnosis or use of psychotropic medications—at baseline. In this way, we showed the temporality of the association between pain at baseline and onset of significant depressive symptoms at follow-up. By designing the study in this way, we aimed to rule out reverse causality in the pain—depressive symptoms relationship. However, participants with subclinical depression at baseline may have been included as well as those with previous resolved depression that remained undiagnosed. Yet, our sensitivity analyses showed a dose–response association between greater pain intensity at baseline and greater risk of onset of significant depressive symptoms at follow-up among men with no or only one depressive symptom at baseline (thus very unlikely to have subclinical depression). A further strength of our cross-national study is the large and diverse sample of participants; we included men and women, middle-aged and older adults, adults with and those without mobility or functional impairment, adults with and those without symptoms of loneliness, across 14 European countries and Israel. The inclusion of participants from many countries is highly relevant, in view of the huge cross-national variation in depressive symptoms [2–4]. We presented age-stratified findings to facilitate a direct comparison with other studies focusing only on middle-aged or older adults. A further strength of our study is the adjustment for several potential confounders and mediators of the pain—depressive symptoms relationship. Moreover, our study explored the pain –depressive symptoms association in a community-dwelling general population, thus complementing previous studies focusing only on populations with specific diseases such as osteoarthritis [50] and highlighting that pain and depressive symptoms were widespread.

Our study also has some limitations. First, depressive symptoms were assessed using a self-reported questionnaire, the EURO-D scale, rather than a clinical interview. Reporting bias, especially underreporting of depressive symptoms, may occur. However, the EURO-D scale is easy to administer and has been widely used and validated to compare depressive symptoms in adults across European and non-European countries [33–35]. Second, our observational design cannot prove causality in the association between pain intensity and depressive symptoms. We could not state that pain caused the onset of significant depressive symptoms or that preventing or relieving pain would reduce the risk of developing clinical depression. Further interventional studies are needed to assess whether better pain management may prevent depression. Third, participants who were lost to follow-up between Wave 5 and 6 were more likely to be men and have moderate or severe pain at baseline. This may have led to underestimation of our associations, especially in men. Fourth, we did not explore how fluctuations in pain intensity or treatment of pain may have modulated the onset of depressive symptoms. However, pain tends to be persistent in middle-aged and older adults [26]. Fifth, we did not assess aspects of pain such as its unpredictability or impact on sleep, which might also affect mood. Sixth, depressive symptoms were assessed at baseline and follow-up and not in the interval between baseline and follow-up. Our study may have missed a small proportion of participants who developed significant depressive symptoms that then resolved in this interval. However, this was likely a small number of cases, leading to minimal underestimation of our associations. Seventh, our inclusion criteria may limit the generalizability of our findings. In particular, our findings cannot be extrapolated to adults living in institutions or those with cognitive impairment. However, our study population was very diverse and comprised adults from 14 countries, with different levels of educational attainment, those with good and those with poor self-rated health, those with and those without mobility and functional impairment. Finally, we cannot exclude residual confounding in our estimates of associations between baseline pain intensity and subsequent risk of significant depressive symptoms.

Our study has clinical relevance and implications. It confirmed the high prevalence of both pain and depressive symptoms among community-dwelling adults in European countries and Israel. Moreover, it supported the hypothesis that pain may precede the onset of depression. Pain is often undertreated, especially in older adults, for concerns about medication side effects and polypharmacy [28]. Further interventional studies should explore whether pharmacological as well as non-pharmacological pain management strategies may reduce the burden of depression in adults.

In conclusion, our study showed that greater pain intensity was prospectively associated with higher risk of onset of significant depressive symptoms in community-dwelling adults without depression at baseline. Moreover, pain and significant depressive symptoms more frequently co-occurred in women than men, yet the associations between pain and depressive symptoms were consistent in both sexes and more accentuated in men. Qualitative research should explore the psychological and sociocultural factors underlying this difference in the strength of the pain – depression association between men and women.

### Supplementary Information

Below is the link to the electronic supplementary material.

Supplementary file1 (PDF 576 KB)

## Data Availability

Data are available upon request from the SHARE website (see http://www.share-project.org/data-access/user-registration.html).
