# Supplementary material for: Association between pain intensity and depressive symptoms in community-dwelling adults: longitudinal findings from the Survey of Health, Ageing and Retirement in Europe (SHARE)
Source: Eur Geriatr Med. 2023 Jul 14;14(5):1111–24. doi: 10.1007/s41999-023-00835-5 (PMC10587243; doi:10.1007/s41999-023-00835-5)
Supplement: Supplementary file 1 — Supplementary file1 (PDF 576 KB) [file 41999_2023_835_MOESM1_ESM.pdf]

Association between pain intensity and depressive symptoms in community-dwelling adults: longitudinal findings from the Survey of Health, Ageing and Retirement in Europe (SHARE)

Journal: European Geriatric Medicine

Giulia Ogliari[1], Jesper Ryg[2,3], Karen Andersen-Ranberg[2,3,4], Lasse Lybecker Scheel-Hincke[4], Jemima T. Collins[1,5], Alison Cowley[5,6], Claudio Di Lorito[7], Vicky Booth[5,6], Roelof A.J. Smit[8], Ralph K. Akyea[9], Nadeem Qureshi[9], David A. Walsh[10,11], Rowan H. Harwood[1,5,12], Tahir Masud[1,12]

1. Department of Health Care for Older People (HCOP), Queen's Medical Centre, Nottingham University Hospitals NHS Trust, Nottingham, UK
2. Department of Geriatric Medicine, Odense University Hospital, Odense, Denmark
3. Geriatric Research Unit, Department of Clinical Research, University of Southern Denmark, Odense, Denmark
4. Unit for Epidemiology, Biostatistics and Biodemography, Department of Public Health, University of Southern Denmark, 5000 Odense, Denmark
5. University of Nottingham, Nottingham, UK
6. Research & Innovation, Nottingham University Hospitals NHS Trust, Nottingham, UK
7. Division of Primary Care and Population Health, University College London, London, UK
8. Novo Nordisk Foundation Center for Basic Metabolic Research, University of Copenhagen, Faculty of Health and Medical Sciences, Blegdamsvej 3B, Building 7 (Maersk Tower), DK-2200 Copenhagen, Denmark
9. Primary Care Stratified Medicine, School of Medicine, University of Nottingham, Nottingham, UK
10. Pain Centre Versus Arthritis, University of Nottingham, Nottingham, UK
11. Sherwood Forest Hospitals NHS Foundation Trust, Sutton-in-Ashfield, UK
12. NIHR Applied Research Collaboration – East Midlands, Nottingham, UK

Corresponding Author: Rowan H. Harwood, Fellow, Department of Health Care for Older People (HCOP), Queen's Medical Centre, Nottingham University Hospitals NHS Trust, Derby Road, Nottingham, Nottinghamshire, NG7 2UH, UK, phone: +44 (0)115 924 9924, e-mail: rowan.harwood@nottingham.ac.uk

e-mail of first Author: giulia.ogliari@virgilio.it and Giulia.Ogliari1@nottingham.ac.uk

Supplementary Table 1 Characteristics at baseline (Wave 5) of participants who were included in the final sample and those who were excluded between Wave 5 and 6

	Excluded at follow-up (n = 8,727)	Final sample (n = 28,515)	P value
Age (years), mean (SD)	65.5 (10.0)	65.4 (9.0)	0.376
Women, n (%)	4,091 (46.9)	14,360 (50.4)	< 0.001
Intensity of pain, n (%)			
No pain	5,856 (67.1)	19,028 (66.7)	0.005
Mild pain	751 (8.6)	2,803 (9.8)	
Moderate pain	1,657 (19.0)	5,253 (18.4)	
Severe pain	463 (5.3)	1,431 (5.0)	
Educational level, n (%)			
None or pre-primary	398 (4.6)	861 (3.0)	< 0.001
Primary	1,309 (15.0)	3,949 (13.8)	
Lower secondary	1,479 (16.9)	4,515 (15.8)	
Upper secondary	3,103 (35.6)	10,087 (35.4)	
Post-secondary non-tertiary	399 (4.6)	1,471 (5.2)	
First stage of tertiary	1,883 (21.6)	7,264 (25.5)	
Second stage of tertiary	115 (1.3)	285 (1.0)	
Still in school or other	41 (0.5)	83 (0.3)	
Fair or poor self-rated health, n (%)	2,390 (27.4)	6,922 (24.3)	< 0.001
BMI category, n (%)			
Underweight	87 (1.0)	257 (0.9)	0.003
Normal weight	3,359 (38.5)	10,445 (36.6)	
Overweight	3,664 (42.0)	12,141 (42.6)	
With obesity	1,617 (18.5)	5,672 (19.9)	
Co-morbidities, n (%)			
Heart attack	750 (8.6)	2,507 (8.8)	0.567
Hypertension	3,152 (36.1)	10,633 (37.3)	0.047

High cholesterol	1,855 (21.3)	6,038 (21.2)	0.871
Stroke	230 (2.6)	651 (2.3)	0.058
Diabetes	910 (10.4)	3,069 (10.8)	0.375
Chronic lung disease	357 (4.1)	1,145 (4.0)	0.754
Cancer	451 (5.2)	1,270 (4.5)	0.005
Parkinson's disease	44 (0.5)	104 (0.4)	0.070
Hip fracture	124 (1.4)	345 (1.2)	0.122
Other fracture	443 (5.1)	1,410 (4.9)	0.621
Rheumatoid arthritis	549 (6.3)	1,809 (6.3)	0.858
Osteoarthritis / other rh.	1,194 (13.7)	4,113 (14.4)	0.083
Drugs, n (%)			
For joint pain	1,028 (11.8)	3,096 (10.9)	0.016
For other pain	699 (8.0)	2,133 (7.5)	0.103
For inflammation*	200 (2.3)	634 (2.2)	0.706
Lives alone, n (%)	1,459 (16.7)	5,290 (18.6)	< 0.001
Physical inactivity, n (%)	684 (7.8)	1,536 (5.4)	< 0.001
Loneliness score, n (%)			
3 (not lonely)	6,273 (71.9)	20,574 (72.2)	0.123
4	1,420 (16.3)	4,637 (16.3)	
5	557 (6.4)	1,885 (6.6)	
6	311 (3.6)	999 (3.5)	
7	101 (1.2)	277 (1.0)	
8	34 (0.4)	76 (0.3)	
9 (very lonely)	31 (0.4)	67 (0.2)	
Mobility impairment	1,278 (14.6)	3,616 (12.7)	< 0.001
ADL impairment	489 (5.6)	1,296 (4.5)	< 0.001
IADL impairment	831 (9.5)	2,068 (7.3)	< 0.001
EURO-D score, n (%)			
0	2,706 (31.0)	9,122 (32.0)	0.161
1	2,574 (29.5)	8,488 (29.8)	
2	2,013 (23.1)	6,320 (22.2)	

3	1,434 (16.4)	4,585 (16.1)

Abbreviation: n: number; SD: standard deviation; other rh.: other rheumatism; ADL: activities of daily living; IADL: instrumental activities of daily living. *Drugs for inflammation: drugs for suppressing inflammation (only glucocorticoids or steroids). P-values were computed by Pearson's chi-square for categorical variables and by ANOVA for age (continuous variable).

Supplementary Table 2 Study population at baseline by country of residence and pain intensity

	All (n = 28,515)	No pain (n = 19,028)	Mild pain (n = 2,803)	Moderate pain (n = 5,253)	Severe pain (n = 1,431)	P value
Austria, n (%)	1,946	1,394 (71.6)	107 (5.5)	367 (18.9)	78 (4.0)	< 0.001
Germany, n (%)	2,841	2,021 (71.1)	131 (4.6)	488 (17.2)	201 (7.1)	
Sweden, n (%)	2,446	1,704 (69.7)	256 (10.5)	409 (16.7)	77 (3.1)	
Spain, n (%)	2,621	1,832 (69.9)	213 (8.1)	442 (16.9)	134 (5.1)	
Italy, n (%)	2,041	1,172 (57.4)	354 (17.3)	417 (20.4)	98 (4.8)	
France, n (%)	1,773	884 (49.9)	247 (13.9)	459 (25.9)	183 (10.3)	
Denmark, n (%)	2,452	1,841 (75.1)	195 (8.0)	324 (13.2)	92 (3.8)	
Switzerland, n (%)	1,871	1,494 (79.9)	114 (6.1)	222 (11.9)	41 (2.2)	
Belgium, n (%)	2,439	1,688 (69.2)	257 (10.5)	387 (15.9)	107 (4.4)	
Israel, n (%)	837	579 (69.2)	85 (10.2)	131 (15.7)	42 (5.0)	
Czech Republic, n (%)	2,683	1,672 (62.3)	388 (14.5)	510 (19.0)	113 (4.2)	
Luxembourg, n (%)	660	441 (66.8)	60 (9.1)	118 (17.9)	41 (6.2)	
Slovenia, n (%)	1,489	855 (57.4)	123 (8.3)	408 (27.4)	103 (6.9)	
Estonia, n (%)	2,416	1,451 (60.1)	273 (11.3)	571 (23.6)	121 (5.0)	

Abbreviation: n: number. Please, note that percentages are row percentages (they indicate the proportion of participants within each country who reported no pain, mild pain, moderate pain and severe pain, respectively. P-value was computed by Pearson's chi-square.

Supplementary Table 3 Study population at baseline by sex, country of residence and pain intensity

	All	No pain	Mild pain	Moderate pain	Severe pain	P value
Men						
All men, n (%)	14,155	9,961 (70.4)	1,352 (9.6)	2,248 (15.9)	594 (4.2)	< 0.001
Austria, n (%)	875	653 (74.6)	45 (5.1)	153 (17.5)	24 (2.7)	
Germany, n (%)	1,516	1,123 (74.1)	68 (4.5)	237 (15.6)	88 (5.8)	
Sweden, n (%)	1,266	939 (74.2)	117 (9.2)	173 (13.7)	37 (2.9)	
Spain, n (%)	1,400	1,070 (76.4)	104 (7.4)	179 (12.8)	47 (3.4)	
Italy, n (%)	1,102	712 (64.6)	168 (15.2)	178 (16.2)	44 (4.0)	
France, n (%)	887	470 (53.0)	127 (14.3)	213 (24.0)	77 (8.7)	
Denmark, n (%)	1,210	951 (78.6)	96 (7.9)	136 (11.2)	27 (2.2)	
Switzerland, n (%)	946	783 (82.8)	53 (5.6)	93 (9.8)	17 (1.8)	
Belgium, n (%)	1,273	920 (72.3)	132 (10.4)	165 (13.0)	56 (4.4)	
Israel, n (%)	394	285 (72.3)	43 (10.9)	52 (13.2)	14 (3.6)	
Czech Republic, n (%)	1,222	793 (64.9)	175 (14.3)	210 (17.2)	44 (3.6)	
Luxembourg, n (%)	348	239 (68.7)	39 (11.2)	49 (14.1)	21 (6.0)	
Slovenia, n (%)	707	433 (61.2)	54 (7.6)	175 (24.8)	45 (6.4)	
Estonia, n (%)	1,009	590 (58.5)	131 (13.0)	235 (23.3)	53 (5.3)	
Women						
All women, n (%)	14,360	9,067 (63.1)	1,451 (10.1)	3,005 (20.9)	837 (5.8)	< 0.001
Austria, n (%)	1,071	741 (69.2)	62 (5.8)	214 (20.0)	54 (5.0)	

Germany, n (%)	1,325	898 (67.8)	63 (4.8)	251 (18.9)	113 (8.5)
Sweden, n (%)	1,180	765 (64.8)	139 (11.8)	236 (20.0)	40 (3.4)
Spain, n (%)	1,221	762 (62.4)	109 (8.9)	263 (21.5)	87 (7.1)
Italy, n (%)	939	460 (49.0)	186 (19.8)	239 (25.5)	54 (5.8)
France, n (%)	886	414 (46.7)	120 (13.5)	246 (27.8)	106 (12.0)
Denmark, n (%)	1,242	890 (71.7)	99 (8.0)	188 (15.1)	65 (5.2)
Switzerland, n (%)	925	711 (76.9)	61 (6.6)	129 (13.9)	24 (2.6)
Belgium, n (%)	1,166	768 (65.9)	125 (10.7)	222 (19.0)	51 (4.4)
Israel, n (%)	443	294 (66.4)	42 (9.5)	79 (17.8)	28 (6.3)
Czech Republic, n (%)	1,461	879 (60.2)	213 (14.6)	300 (20.5)	69 (4.7)
Luxembourg, n (%)	312	202 (64.7)	21 (6.7)	69 (22.1)	20 (6.4)
Slovenia, n (%)	782	422 (54.0)	69 (8.8)	233 (29.8)	58 (7.4)
Estonia, n (%)	1,407	861 (61.2)	142 (10.1)	336 (23.9)	68 (4.8)

Abbreviation: n: number. Please, note that percentages are row percentages (they indicate the proportion of men / women within each country who reported no pain, mild pain, moderate pain and severe pain, respectively). P-values were computed by Pearson's chi-square and refer to the cross-country variation in distribution of pain intensity categories.

Supplementary Table 4 Characteristics of study population at baseline, by sex

	All (n = 28,515)	Men (n = 14,155)	Women (n = 14,360)	P value
Age (years), mean (SD)	65.4 (9.0)	65.8 (8.9)	65.0 (9.0)	< 0.001
Intensity of pain, n (%)				
No pain	19,028 (66.7)	9,961 (70.4)	9,067 (63.1)	< 0.001
Mild pain	2,803 (9.8)	1,352 (9.6)	1,451 (10.1)	
Moderate pain	5,253 (18.4)	2,248 (15.9)	3,005 (20.9)	
Severe pain	1,431 (5.0)	594 (4.2)	837 (5.8)	
Educational level, n (%)				
None or pre-primary	861 (3.0)	447 (3.2)	414 (2.9)	< 0.001
Primary	3,949 (13.8)	1,851 (13.1)	2,098 (14.6)	
Lower secondary	4,515 (15.8)	2,126 (15.0)	2,389 (16.6)	
Upper secondary	10,087 (35.4)	5,035 (35.6)	5,052 (35.2)	
Post-secondary non-tertiary	1,471 (5.2)	713 (5.0)	758 (5.3)	
First stage of tertiary	7,264 (25.5)	3,754 (26.5)	3,510 (24.4)	
Second stage of tertiary	285 (1.0)	190 (1.3)	95 (0.7)	
Still in school or other	83 (0.3)	39 (0.3)	44 (0.3)	
Fair or poor self-rated health, n (%)	6,922 (24.3)	3,520 (24.9)	3,402 (23.7)	0.020
BMI category, n (%)				
Underweight	257 (0.9)	41 (0.3)	216 (1.5)	< 0.001
Normal weight	10,445 (36.6)	4,363 (30.8)	6,082 (42.4)	
Overweight	12,141 (42.6)	6,951 (49.1)	5,190 (36.1)	
With obesity	5,672 (19.9)	2,800 (19.8)	2,872 (20.0)	
Co-morbidities, n (%)				
Heart attack	2,507 (8.8)	1,584 (11.2)	923 (6.4)	< 0.001
Hypertension	10,633 (37.3)	5,384 (38.0)	5,249 (36.6)	0.010
High cholesterol	6,038 (21.2)	3,079 (21.8)	2,959 (20.6)	0.018
Stroke	651 (2.3)	384 (2.7)	267 (1.9)	< 0.001

Diabetes	3,069 (10.8)	1,789 (12.6)	1,280 (8.9)	< 0.001
Chronic lung disease	1,145 (4.0)	626 (4.4)	519 (3.6)	0.001
Cancer	1,270 (4.5)	623 (4.4)	647 (4.5)	0.669
Parkinson’s disease	104 (0.4)	67 (0.5)	37 (0.3)	0.003
Hip fracture	345 (1.2)	179 (1.3)	166 (1.2)	0.402
Other fracture	1,410 (4.9)	707 (5.0)	703 (4.9)	0.699
Rheumatoid arthritis	1,809 (6.3)	651 (4.6)	1,158 (8.1)	< 0.001
Osteoarthritis / other rh.	4,113 (14.4)	1,616 (11.4)	2,497 (17.4)	< 0.001
Drugs, n (%)				
For joint pain	3,096 (10.9)	1,197 (8.5)	1,899 (13.2)	< 0.001
For other pain	2,133 (7.5)	778 (5.5)	1,355 (9.4)	< 0.001
For inflammation*	634 (2.2)	251 (1.8)	383 (2.7)	< 0.001
Lives alone, n (%)	5,290 (18.6)	1,818 (12.8)	3,472 (24.2)	< 0.001
Physical inactivity, n (%)	1,536 (5.4)	700 (4.9)	836 (5.8)	0.001
Loneliness score, n (%)				
3 (not lonely)	20,574 (72.2)	10,434 (73.7)	10,140 (70.6)	< 0.001
4	4,637 (16.3)	2,198 (15.5)	2,439 (17.0)	
5	1,885 (6.6)	908 (6.4)	977 (6.8)	
6	999 (3.5)	438 (3.1)	561 (3.9)	
7	277 (1.0)	116 (0.8)	161 (1.1)	
8	76 (0.3)	35 (0.2)	41 (0.3)	
9 (very lonely)	67 (0.2)	26 (0.2)	41 (0.3)	
Mobility impairment	3,616 (12.7)	1,323 (9.3)	2,293 (16.0)	< 0.001
ADL impairment	1,296 (4.5)	661 (4.7)	635 (4.4)	0.315
IADL impairment	2,068 (7.3)	781 (5.5)	1,287 (9.0)	< 0.001
EURO-D score, n (%)				
0	9,122 (32.0)	5,104 (36.1)	4,018 (28.0)	< 0.001
1	8,488 (29.8)	4,349 (30.7)	4,139 (28.8)	
2	6,320 (22.2)	2,864 (20.2)	3,456 (24.1)	
3	4,585 (16.1)	1,838 (13.0)	2,747 (19.1)	

Abbreviation: n: number; SD: standard deviation; BMI: body mass index; other rh.: other rheumatism; ADL: activities of daily living; IADL: instrumental activities of daily living. *Drugs for inflammation: drugs for suppressing inflammation (only glucocorticoids or steroids). P-values were computed by Pearson's chi-square for categorical variables and by ANOVA for age (continuous variable).

Supplementary Table 5 Significant depressive symptoms at follow-up by sex and country of residence

	All	Significant depressive symptoms (EURO-D score ≥4)	P value
Men			
All men, n (%)	14,155	1,451 (10.3)	< 0.001
Austria, n (%)	875	71 (8.1)	
Germany, n (%)	1,516	143 (9.4)	
Sweden, n (%)	1,266	85 (6.7)	
Spain, n (%)	1,400	157 (11.2)	
Italy, n (%)	1,102	156 (14.2)	
France, n (%)	887	113 (12.7)	
Denmark, n (%)	1,210	64 (5.3)	
Switzerland, n (%)	946	67 (7.1)	
Belgium, n (%)	1,273	124 (9.7)	
Israel, n (%)	394	50 (12.7)	
Czech Republic, n (%)	1,222	118 (9.7)	
Luxembourg, n (%)	348	44 (12.6)	
Slovenia, n (%)	707	86 (12.2)	
Estonia, n (%)	1,009	173 (17.1)	
Women			
All women, n (%)	14,360	2,417 (16.8)	< 0.001
Austria, n (%)	1,071	155 (14.5)	
Germany, n (%)	1,325	210 (15.8)	
Sweden, n (%)	1,180	153 (13.0)	
Spain, n (%)	1,221	234 (19.2)	
Italy, n (%)	939	210 (22.4)	
France, n (%)	886	211 (23.8)	
Denmark, n (%)	1,242	123 (9.9)	
Switzerland, n (%)	925	118 (12.8)	

Belgium, n (%)	1,166	201 (17.2)
Israel, n (%)	443	78 (17.6)
Czech Republic, n (%)	1,461	225 (15.4)
Luxembourg, n (%)	312	58 (18.6)
Slovenia, n (%)	782	127 (16.2)
Estonia, n (%)	1,407	314 (22.3)

Abbreviation: n: number. Please, note that percentages are row percentages (they indicate the proportion of men / women within each country who reported significant depressive symptoms at follow-up, respectively). P-values were computed by Pearson's chi-square and refer to the cross-country variation in proportion of men / women, respectively, with significant depressive symptoms at follow-up.

Supplementary Table 6 Longitudinal association between pain intensity at baseline and significant depressive symptoms at follow-up, in participants without mobility or ADL or IADL impairment at baseline (n = 23,716)

	**All (n = 23,716)**		**Men (n = 12,264)**		**Women (n = 11,452)**	
	**OR [95% CI]**	**P value**	**OR [95% CI]**	**P value**	**OR [95% CI]**	**P value**
**Model 1**						
No pain	1 (ref)		1 (ref)		1 (ref)	
Mild pain	1.52 [1.34; 1.73]	< 0.001	1.62 [1.33; 1.97]	< 0.001	1.46 [1.23; 1.72]	< 0.001
Moderate pain	1.74 [1.57; 1.93]	< 0.001	2.02 [1.72; 2.38]	< 0.001	1.58 [1.38; 1.81]	< 0.001
Severe pain	2.14 [1.75; 2.63]	< 0.001	2.19 [1.58; 3.05]	< 0.001	2.11 [1.63; 2.73]	< 0.001
**Model 2**						
No pain	1 (ref)		1 (ref)		1 (ref)	
Mild pain	1.34 [1.17; 1.53]	< 0.001	1.42 [1.15; 1.74]	0.001	1.28 [1.08; 1.53]	0.005
Moderate pain	1.36 [1.21; 1.54]	< 0.001	1.57 [1.31; 1.90]	< 0.001	1.24 [1.07; 1.45]	0.006
Severe pain	1.48 [1.19; 1.84]	0.001	1.42 [0.99; 2.03]	0.057	1.54 [1.16; 2.04]	0.003
**Model 3**						
No pain	1 (ref)		1 (ref)		1 (ref)	
Mild pain	1.32 [1.16; 1.51]	< 0.001	1.39 [1.13; 1.71]	0.002	1.28 [1.07; 1.52]	0.007
Moderate pain	1.33 [1.18; 1.50]	< 0.001	1.52 [1.26; 1.84]	< 0.001	1.22 [1.05; 1.43]	0.011
Severe pain	1.46 [1.17; 1.82]	0.001	1.38 [0.96; 1.98]	0.080	1.54 [1.16; 2.04]	0.003

Odds ratios and 95% confidence intervals were calculated by binary logistic regression. Model 1: adjusted for age and sex. Model 2: Model 1 + education, self-rated health, body mass index (BMI) category, heart attack, hypertension, high cholesterol, stroke, diabetes,

chronic lung disease, cancer, Parkinson's, hip fracture, other fractures, rheumatoid arthritis, osteoarthritis or other rheumatism, drugs for joint pain, drugs for other pain, drugs for suppressing inflammation (only glucocorticoids or steroids), living alone, country. Model 3: Model 2 + physical inactivity, mobility impairment, Activities of Daily Living (ADL) impairment, Instrumental ADL (IADL) impairment, loneliness.

Supplementary Table 7 Longitudinal association between pain intensity at baseline and significant depressive symptoms at follow-up, in participants without loneliness at baseline (n = 20,574)

	**All (n = 20,574)**		**Men (n = 10,434)**		**Women (n = 10,140)**	
	**OR [95% CI]**	**P value**	**OR [95% CI]**	**P value**	**OR [95% CI]**	**P value**
**Model 1**						
No pain	1 (ref)		1 (ref)		1 (ref)	
Mild pain	1.38 [1.19; 1.60]	< 0.001	1.46 [1.16; 1.84]	0.001	1.33 [1.10; 1.61]	0.003
Moderate pain	1.91 [1.72; 2.12]	< 0.001	2.08 [1.75; 2.47]	< 0.001	1.82 [1.59; 2.08]	< 0.001
Severe pain	2.44 [2.06; 2.88]	< 0.001	2.71 [2.05; 3.58]	< 0.001	2.30 [1.87; 2.84]	< 0.001
**Model 2**						
No pain	1 (ref)		1 (ref)		1 (ref)	
Mild pain	1.20 [1.03; 1.39]	0.021	1.29 [1.01; 1.64]	0.041	1.14 [0.94; 1.39]	0.193
Moderate pain	1.42 [1.25; 1.60]	< 0.001	1.64 [1.34; 2.00]	< 0.001	1.30 [1.11; 1.53]	0.001
Severe pain	1.52 [1.25; 1.84]	< 0.001	1.81 [1.32; 2.49]	< 0.001	1.37 [1.07; 1.75]	0.013
**Model 3**						
No pain	1 (ref)		1 (ref)		1 (ref)	
Mild pain	1.18 [1.01; 1.37]	0.036	1.27 [0.99; 1.61]	0.058	1.12 [0.92; 1.37]	0.264
Moderate pain	1.33 [1.17; 1.51]	< 0.001	1.52 [1.24; 1.86]	< 0.001	1.23 [1.05; 1.44]	0.011
Severe pain	1.32 [1.08; 1.62]	0.006	1.51 [1.09; 2.10]	0.013	1.22 [0.95; 1.57]	0.126

Odds ratios and 95% confidence intervals were calculated by binary logistic regression. Model 1: adjusted for age and sex. Model 2: Model 1 + education, self-rated health, body mass index (BMI) category, heart attack, hypertension, high cholesterol, stroke, diabetes,

chronic lung disease, cancer, Parkinson's, hip fracture, other fractures, rheumatoid arthritis, osteoarthritis or other rheumatism, drugs for joint pain, drugs for other pain, drugs for suppressing inflammation (only glucocorticoids or steroids), living alone, country. Model 3: Model 2 + physical inactivity, mobility impairment, Activities of Daily Living (ADL) impairment, Instrumental ADL (IADL) impairment, loneliness.

Supplementary Table 8 Longitudinal association between pain intensity at baseline and significant depressive symptoms at follow-up, in participants with EURO-D score 0-1 at baseline (n = 17,610)

	**All (n = 17,610)**		**Men (n = 9,453)**		**Women (n = 8,157)**	
	**OR [95% CI]**	**P value**	**OR [95% CI]**	**P value**	**OR [95% CI]**	**P value**
**Model 1**						
No pain	1 (ref)		1 (ref)		1 (ref)	
Mild pain	1.47 [1.24; 1.75]	< 0.001	1.60 [1.22; 2.08]	0.001	1.39 [1.10; 1.75]	0.005
Moderate pain	1.71 [1.49; 1.96]	< 0.001	1.86 [1.50; 2.31]	< 0.001	1.62 [1.36; 1.93]	< 0.001
Severe pain	2.32 [1.84; 2.94]	< 0.001	2.48 [1.70; 3.63]	< 0.001	2.23 [1.66; 3.00]	< 0.001
**Model 2**						
No pain	1 (ref)		1 (ref)		1 (ref)	
Mild pain	1.23 [1.03; 1.48]	0.025	1.47 [1.11; 1.94]	0.007	1.08 [0.85; 1.38]	0.542
Moderate pain	1.22 [1.04; 1.43]	0.015	1.60 [1.24; 2.04]	< 0.001	1.03 [0.84; 1.27]	0.752
Severe pain	1.45 [1.11; 1.89]	0.006	1.90 [1.24; 2.91]	0.003	1.22 [0.87; 1.72]	0.252
**Model 3**						
No pain	1 (ref)		1 (ref)		1 (ref)	
Mild pain	1.23 [1.02; 1.48]	0.027	1.44 [1.09; 1.91]	0.010	1.08 [0.85; 1.38]	0.527
Moderate pain	1.13 [0.96; 1.33]	0.130	1.47 [1.14; 1.89]	0.003	0.97 [0.78; 1.19]	0.753
Severe pain	1.30 [0.99; 1.71]	0.061	1.66 [1.07; 2.59]	0.024	1.10 [0.77; 1.56]	0.608

Odds ratios and 95% confidence intervals were calculated by binary logistic regression. Model 1: adjusted for age and sex. Model 2: Model 1 + education, self-rated health, body mass index (BMI) category, heart attack, hypertension, high cholesterol, stroke, diabetes,

chronic lung disease, cancer, Parkinson's, hip fracture, other fractures, rheumatoid arthritis, osteoarthritis or other rheumatism, drugs for joint pain, drugs for other pain, drugs for suppressing inflammation (only glucocorticoids or steroids), living alone, country. Model 3: Model 2 + physical inactivity, mobility impairment, Activities of Daily Living (ADL) impairment, Instrumental ADL (IADL) impairment, loneliness.

Supplementary Table 9 Number of participants in a cross-table showing pain intensity at baseline (Wave 5) by pain intensity follow-up (Wave 6) (in 28,502 participants)

		Pain intensity at follow-up (Wave 6)			
		**No pain**	**Mild pain**	**Moderate pain**	**Severe pain**
**Pain intensity at baseline (Wave 5)**	**No pain**	14,558	1,709	2,262	495
	**Mild pain**	1,313	562	792	133
	**Moderate pain**	1,666	659	2,313	611
	**Severe pain**	330	115	496	488

This cross-table shows 28,502 participants who had data on pain intensity at both baseline (Wave 5) and follow-up (Wave 6). We excluded 13 participants from the analyses due to missing data on pain intensity at follow-up. We classified 6,002 (21.1%) participants as having worsening pain intensity from baseline to follow-up [in detail, no pain -> mild pain: 1,709; no pain -> moderate pain: 2,262; no pain -> severe pain: 495; mild pain -> moderate pain: 792; mild pain -> severe pain: 133; moderate pain -> severe pain: 611 participants].

Supplementary Table 10 Longitudinal association between number of depressive symptoms at baseline (EURO-D score) and likelihood of worsening pain intensity from baseline to follow-up (in 28,502 participants)

	**All (n = 28,502)**	
	**B-coefficient [95% CI for B-coefficient]**	**P value**
**Model 1**		
EURO-D score at baseline	1.09 [1.07; 1.12]	< 0.001
**Model 2 without medications**		
EURO-D score at baseline	1.06 [1.03; 1.09]	< 0.001
**Model 2 with medications**		
EURO-D score at baseline	1.06 [1.03; 1.09]	< 0.001
**Model 3**		
EURO-D score at baseline	1.07 [1.04; 1.10]	< 0.001

We investigated the longitudinal association between number of depressive symptoms at baseline (EURO-D score at baseline) (determinant, continuous variable) and likelihood of having worsening pain intensity from baseline to follow-up (binary outcome). We used binary logistic regression models. In total, 6,002 out of 28,502 participants had worsening pain intensity from baseline to follow-up. Abbreviation: CI: confidence interval. Model 1: adjusted for age and sex. Model 2 without medications: Model 1 + education, self-rated health, body mass index (BMI) category, heart attack, hypertension, high cholesterol, stroke, diabetes, chronic lung disease, cancer, Parkinson's, hip fracture, other fractures, rheumatoid arthritis, osteoarthritis or other rheumatism, living alone, country. Model 2 with medications: Model 2 without medications + drugs for joint pain, drugs for other pain, drugs for suppressing inflammation (only glucocorticoids or steroids). Model 3: Model 2 + physical inactivity, mobility impairment, Activities of Daily Living (ADL) impairment, Instrumental ADL (IADL) impairment, loneliness.
